# Accurate Natural Trail Detection Using a Combination of a Deep Neural Network and Dynamic Programming

**DOI:** 10.3390/s18010178

**Published:** 2018-01-10

**Authors:** Shyam Prasad Adhikari, Changju Yang, Krzysztof Slot, Hyongsuk Kim

**Affiliations:** 1 Division of Electronics Engineering, Chonbuk National University, Jeonju 567-54896, Korea; all.shyam@gmail.com (S.P.A); ychangju@jbnu.ac.kr (C.J.Y); 2 Institute of Applied Computer Science, Lodz University of Technology, Stefanowskiego 18/22, 90-924 Lodz, Poland; krzysztof.slot@p.lodz.pl; 3 Intelligent Robot Research Center of Chonbuk National University, Chonbuk National University, Jeonju 567-54896, Korea

**Keywords:** deep neural networks, trail segmentation, trail following, dynamic programming

## Abstract

This paper presents a vision sensor-based solution to the challenging problem of detecting and following trails in highly unstructured natural environments like forests, rural areas and mountains, using a combination of a deep neural network and dynamic programming. The deep neural network (DNN) concept has recently emerged as a very effective tool for processing vision sensor signals. A patch-based DNN is trained with supervised data to classify fixed-size image patches into “trail” and “non-trail” categories, and reshaped to a fully convolutional architecture to produce trail segmentation map for arbitrary-sized input images. As trail and non-trail patches do not exhibit clearly defined shapes or forms, the patch-based classifier is prone to misclassification, and produces sub-optimal trail segmentation maps. Dynamic programming is introduced to find an optimal trail on the sub-optimal DNN output map. Experimental results showing accurate trail detection for real-world trail datasets captured with a head mounted vision system are presented.

## 1. Introduction

Autonomous navigation in highly unstructured environments like man-made trails in forests or mountains is an extremely challenging problem for robots. Humans can navigate through most off-road trails with ease, however the infinite variations present in the natural environment, the absence of structured pathways or distinct lane markings makes the problem of trail navigation extremely difficult for robotic systems. A robotic system capable of autonomously navigating off-road environments would become invaluable aid in several important applications, such as search-and-rescue missions, wilderness monitoring and mapping etc.

The problem of road and lane detection in structured environments like paved roads and highways has been studied extensively in the literature, and has been a crucial enabler towards the realization of autonomous vehicles [1,2,3,4,5,6]. However, detecting trails in off-road environments like forests and mountains which, at times, is challenging even for humans, is significantly more difficult for robots. The problem of off-road trail detection has been approached primarily as a segmentation problem [7,8,9] i.e., how to segment the trail region from surrounding areas. A simplified model of the trail is then fit to the segmented image. Rasmussen et al. [8] used local appearance contrast visual cues and lidar-derived shape cues to segment the trail from the surrounding areas, whereas Santana et al. [9] used image conspicuity to compute a saliency map of an input image to detect the position of the trail. 

Recently deep neural networks (DNNs) have been widely used for various vision-related applications [10,11], and have produced state-of-the-art results in different tasks like object detection and localization [12], image segmentation [13] and depth perception from monocular or stereo images [14,15]. With a focus towards realizing self-driving vehicles, different researchers have successfully applied DNNs for road and lane detection in highways and urban settings. Huval et al. [16] used a variant of DNN for detecting the road and lanes for driving autonomously on highways. Instead of detecting and recognizing objects like lanes, vehicle, pedestrians, etc. [17] used a variant of DNN to map an input image to several driving indicators like distance to lane markings, angle of vehicle with respect to the lane etc. which are fed to a controller for autonomous driving in urban environment. Bojarskiet et al. [18] trained a DNN to directly map the raw pixels of an input image to steering commands for autonomous driving in highway and urban settings. Although the task of trail detection is related to the task of road (lane) detection, majority of methods developed for the latter case rely heavily on road image models that involve several prior knowledge clues, such as presence of expected road markings, road/lane geometry constraints or temporal consistency [5,19]. These priors are utilized to cope with occlusions, shadows, under- and over-exposure or glare, i.e., factors that are common in traffic situations, yet are not necessarily relevant for trail detection. Also, commonly used image representations employ edges as one of the most useful features in road/lane detection [6], however, as edges are poor texture descriptors, they are inappropriate for trail representation. Therefore, even though deep convolutional neural networks have also been considered as a means for road detection [20], due to important differences between the two considered tasks, obtained results are not necessarily meaningful for the case of trail detection with DNN.

DNNs have also been used for autonomous navigation of robots in unstructured natural environments like forest trails. Hadsell et al. [21] used a self-supervised DNN with a stereo module in the loop to classify the terrain in front of the robot as ground or obstacle. The self-supervised learning system used a stereo module that provided supervising class labels for learning a DNN. The class label for each image patch was assigned using a series of heuristics depending on the ground, foot line and the obstacle plane derived from the 3d point cloud. However, in natural environment the trail and the surrounding areas can share the same height; a straightforward use of the 3d point cloud information as supervising teacher for learning provides incorrect labels and hence incorrect learning behavior of the DNN. Given an image of the trail as input Guisti et al. [22] used DNN as a supervised classifier to output only the main direction of a trail compared to the viewing direction of a quadrotor. Similar approach using DNN has been used by Nikolai et al. [23] to estimate the view orientation along with the lateral offsets of a micro aerial vehicle with respect to the trail center. The work of both Guisti et al. and Nikolai et al. estimate the instantaneous heading direction of the trail and do not utilize the information present in the input image that could assist in planning the path for the local segment of the trail visible at that instant. 

In this paper we propose a two-stage pipeline using a combination of DNN and dynamic programming to detect and follow trails in natural environments. In the first stage we train a supervised patch-based DNN to classify each patch in the image as “trail” or “non-trail”, and produce a trail segmentation map for the whole image. As trail and non-trail patches do not exhibit clearly defined shapes or forms, the patch-based classifier is prone to misclassification, and the resultant trail segmentation map is sub-optimal. In the second stage, dynamic programming is used on this sub-optimal trail map to find an optimal trail. In addition to the instantaneous heading direction, the proposed method also computes the local segment of the visible trail.

The rest of the paper is organized as follows: the proposed method for detecting a trail is presented in Section 2 followed by the use of dynamic programming for trail following in Section 3. Experiments conducted to validate the proposed method and the results obtained for real-world trail dataset are presented in Section 4, and are followed by our conclusions in Section 5.

## 2. Patch-Based Deep Neural Network for Trail Segmentation

**Figure 1:**
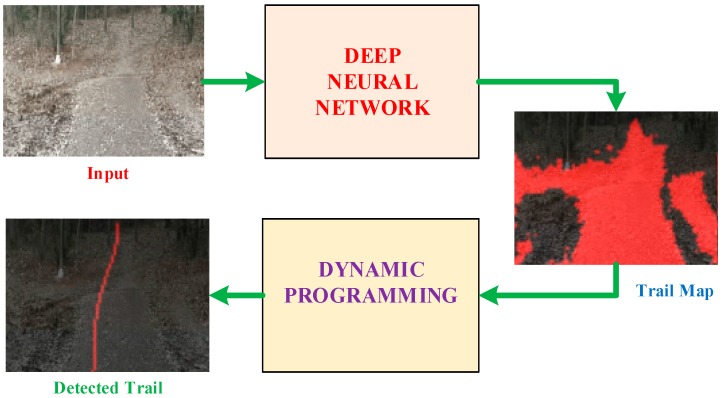
Natural trail detection system using a deep neural network (DNN) and dynamic programming (DP). A DNN trained with supervised data maps the input image into trail and non-trail areas. The starting and the goal point of the local segment are computed using the output from the DNN. DP is then used on the trail map to obtain the local segment of the visible trail.

The proposed method to detect trail in a single image of highly unstructured natural environment is presented in Figure 1.

The core idea is to train a DNN to classify the center pixel of each patch in the image as belonging to trail or not, and obtain a coarse trail segmentation map. The starting point and endpoint for the local segment of the visible trail in the input image are extracted using the resultant trail map and dynamic programming is used on the sub-optimal segmentation map to find an optimal trail line for the visible trail segment. 

Detection of natural trails is a challenging problem due to wide variations in appearance of natural environments, and at times there is no distinct demarcation between the trail and the surrounding areas. It is practically not possible to collect and label a huge dataset that covers all the variations present in natural trail and its surrounding environment. Therefore we restrict our experiments to a subset of the IDSIA forest dataset available at [24]. However, we later show that the proposed approach can be adopted to a completely different trail by fine tuning the DNN with a small subset of data from the new environment.

### 2.1. Dataset

**Figure 2:**
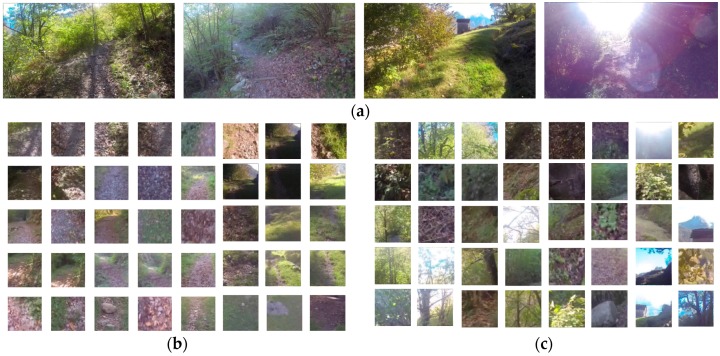
(**a**) Examples of trail images from the IDSIA dataset used for our experiment. The dataset to train deep neural network consists of 100 × 100 RGB patches for the trail and non-trail areas; (**b**) “Trail” patches are extracted from the regions where hikers would walk, but without any distinct boundary or markings; (**c**) “Non-trail” patches are extracted from other surrounding areas in the image.

A subset of the IDSIA forest trail dataset was used to train and test the DNN. The IDSIA forest trail dataset contains images of natural forest trail captured using different cameras and of varying resolution − some are 752 × 480 whereas others are 1280 × 720. We resized all the images to 752 × 480 for our experiments. We use only a subset of the IDSIA dataset for our experiments, namely images from the dataset numbered 001. The images in this folder are captured using three head-mounted cameras oriented in different directions. Out of the images captured from the left, straight and right facing cameras, we use only the images captured with the straight facing camera (from folder named 001/sc) because the trail is not visible in most of the images captured using the other two cameras. The folder “001/sc” contains a total of 3424 images in its three subfolders named “001/sc/GOPR0050”, “001/sc/GP010050” and 001/sc/GP020050”; each containing 1567, 1566 and 299 images, respectively. Each subfolder contains images from different sections of the trail. Images from the subfolder GOPR0050 were used for training and validation, whereas images from subfolder GP010050 and GP020050 were used for testing the network. Several images from the dataset are shown in Figure 2.

The data to train the DNN was prepared by extracting 100 × 100 RGB image patches from the trail images and manually labeling each patch as either “trail” or “non-trail”. Image patches assumed appropriate for hiking were labeled as trail, whereas patches from surrounding areas were labeled as non-trail. Some of the extracted patches from “trail” and the surrounding “non-trail” regions are shown Figure 2b,c, respectively. 

A total of 68,942 patches were extracted from the training folder GOPR0050, out of which 14,936 were trail patches whereas 54,006 were non-trail patches from surrounding areas. 90% of the image patches were used for training the network and the remaining 10% were set aside for validation. The data was augmented during runtime by generating random crops of size 80 × 80 from the original 100 × 100 patches and their corresponding horizontal mirrors. Similarly, a total of 88,060 (17,440 “trail” and 70620 “non-trail”) patches extracted from the folders GP010050 and GP020050 were used for testing the DNN. The number of patches in the trail and non-trail categories is un-balanced in the training as well as the test set. As the trail occupies a smaller area in the image compared to the surrounding areas, the ratio of the trail to non-trail patches in the data reflects the actual ratio of patches that are expected to be present in natural trail images.

### 2.2. Deep Neural Network for Image Patch Classification

A deep neural network is composed of a series of non-linear processing layers stacked on top of each other. Typical layers present in DNN are convolutional, pooling, fully connected and non-linear activation layers. The convolutional layer operates on the local volumes of data through convolutional kernels also called filters to extract feature representations. The pooling layer progressively reduces the spatial size of the feature maps, by pooling maximum activations (in case of max pooling) from non-overlapping regions in the feature maps. This reduces the amount of parameters and computation in the network. The DNN is then trained to map the inputs to their corresponding targets using gradient-descent based learning rules.

#### 2.2.1. Deep Neural Network Architecture

Theoretical guidelines for optimizing deep convolutional network architectures for a given task realization are still missing. Therefore, the approach adopted for this purpose is to experiment with different structures that implement various intuitions. For example, a need for providing a sufficient capacity for correct representation of underlying complex data structures, through ensuring a sufficient amount of filters, amount of scaling steps and organization of a fully connected layer, was at the core of development of AlexNet [11] and ZF Net [25]. Enforcing the same detail of analysis at different scales (the same size of filters at different layers) was a novelty introduced in VGG Net [26]. Reducing complexity of a task to be learned by different layers underlies a concept of incremental learning, proposed in Residual Nets [27].

Natural trails are textural image objects of large variability and diverse structures. Therefore, machine learning becomes clearly an appropriate paradigm for implementing a trail detection algorithm. On the other hand, trail variability and diversity makes it quite difficult to point any particular, preferable network’s architecture for the task realization. As a result, of several possible candidates, the well-known AlexNet DNN model, which is relatively simple and proved successful in recognizing a wide variety of image objects, has been adopted for the presented research.

**Figure 3:**
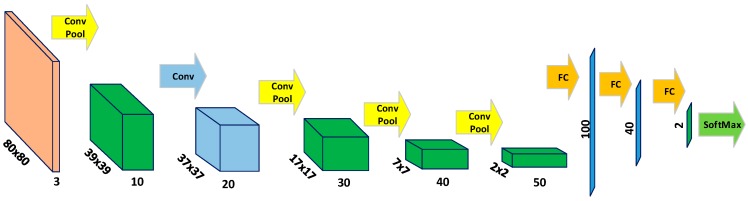
Deep Neural Network architecture. The network is composed of five convolutional layers and three fully connected (FC) layers, with a Softmax classifier on top. The input to the network is a RGB color image patch of size 80 × 80 pixels. The network outputs two numbers corresponding to the probability of the input patch belonging to the trail and non-trail area, respectively.

A deep neural network, as shown in Figure 3, of architecture similar to the flagship AlexNet is used for training our patch classifier to discriminate between the trail and non-trail patches. The DNN consists of eight layers in which the first five layers are convolutional layers followed by three fully connected layers and a softmax function at the output. The input to the DNN is an 80 × 80 RGB color image patch. Max pooling is used after the first, third, fourth and fifth convolutional layers to reduce the spatial size of the feature maps. The neurons in the fully connected (FC) layers receive inputs from all the units in the previous layer and the last FC layer is followed by a softmax function. Given an input image patch, the network outputs two real valued numbers between [0, 1], that can be interpreted as the normalized class probability of the image patch belonging to the “trail” or the surrounding “non-trail” areas.

#### 2.2.2. Deep Neural Network Training

The parameters, θ, of the network are initialized using the Xavier [28] initialization method. The output of the deep convolutional neural network can be interpreted as the model for the conditional distribution over the two classes. The training criterion adopted to maximize the probability of the true category in the training data, D, or equivalently to minimize the negative log-likelihood loss, is the following:

(1) $$l(\theta ,D)=-\sum_{i=0}^{\left|D\right|}\log(P(Y=y^{\left(i\right)}|x^{\left(i\right)},\theta ))$$

where $P(Y=y^{\left(i\right)}|x^{\left(i\right)},\theta )$ is the probability that the input data *x*^(*i*)^ belongs to its true class *y*^(*i*)^. The network was trained in Theano [29] on a GTX 980 GPU using the Adam [30] method with a fixed learning rate of 0.0001 and mini-batch size of 128. Dropout (with *p* = 0.5) was used in the two penultimate fully connected layers and L2 regularization (λ = 0.0001) was implemented to prevent over-fitting.

### 2.3. Fully Convolutional Neural Network for Trail Map Generation

**Figure 4:**
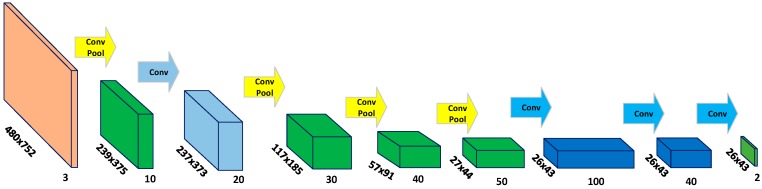
Fully Convolutional Neural Network (FCN) corresponding to the DNN shown in Figure 3. The FCN is obtained by converting the last three fully connected (FC) layers of the DNN to convolutional layers by reshaping the FC layers. The network can process arbitrary sized input images and the output of the network are two score maps corresponding to the trail and the non-trail category, respectively. Given an RGB input of size 480 × 752, the network outputs two feature maps of size 26 × 43 pixels each. Each point in the output map represents the normalized probability of the corresponding image patch belonging to one of the considered categories.

The deep neural network shown in Figure 3 takes fixed-size image patch as input and outputs two scores for the center pixel belonging to trail or non-trail category, respectively. The fully connected layers of the DNN can only process fixed sized inputs, whereas the convolutional layers allow for processing of arbitrary sized inputs. Since, neurons in both the convolutional and fully connected layers compute the dot product of the input with the layer parameters it is always possible to convert the fully connected layer into a convolutional layer. In order to make the network work for images of arbitrary size, the three fully connected layers at the trailing end of the DNN are converted to convolutional layers by introducing appropriate rearrangements. The resulting Fully Convolutional Network (FCN) [31] hence obtained is shown in Figure 4.

**Figure 5:**
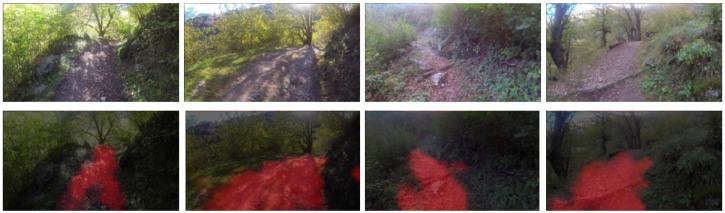
Trail segmentation using DNN. (Top row) Some representative images (resized to 240 × 376) from the test set. (Bottom row) The trail maps obtained using the proposed pipeline are overlaid (26 × 43 maps are up-sampled to 240 × 376) on the corresponding test images. The probabilities that points belong to a trail are coded with intensity of the red component, and a weighted sum with the image pixels is computed.

The FCN can process arbitrary sized input images and outputs two score maps corresponding to the trail and the non-trail category, respectively. The required trail segmentation map is the output map corresponding to the trail class. Each point in this map represents a score for the corresponding image patch in the input image belonging to a trail. The segmentation map of the trail obtained using the above mentioned patch wise classification is noisy. Hence a post processing step is employed on the trail map by using morphological opening to filter out possible small spurious regions and make the trail map smoother. Results of the trail segmentation for some of the images from the test set are shown in Figure 5.

### 2.4. Starting Point and Terminal Row of the Trail

In our experiments we only consider the case where the images are captured with a camera facing straight towards the trail. Once the trail has been segmented from the surrounding areas, we strive to find the starting point and a row of an image where a trail vanishes (referred from now on as a ‘terminal row’) of the local segment of the trail visible in the input without imposing any constraints on a camera position with respect to a trail. The starting point of a trail is determined by computing the center-of-mass of the segmentation map at the bottom row, and the terminal row is the first upper row containing the trail points. Dynamic programming is then used on the trail probability map to find the trail line originating from the starting point towards the terminal row.

## 3. Dynamic Programming for Trail Line Detection

Dynamic programming (DP) is a global optimization method for computing the optimal path between two nodes that is based on the Bellman’s local optimality principle [32]. In our case, we consider each pixel in the trail probability map as a node of a corresponding search graph in order to find a trail line from the starting point to the terminal row. Dynamic programming consists of two phases that gets executed in order to find the lowest-cost path. In the first phase, the minimum cost of visiting any of the graph nodes from the terminal row nodes is computed using a recurrent formula of the general form:

(2) $$c_{ij}^{*}=\underset{k,l}{\min}\{c_{kl}^{*}+d_{kl->ij}+d_{ij}\}$$

where *d_kl__→ij_* denotes the cost of transition from node *kl* to node *ij*, *d_ij_* is the cost associated with node *ij* and $c_{kl}^{*}$ is the minimum cost computed for all the valid predecessors of the node *ij*. In the second phase of the algorithm the lowest cost path originating at the starting point towards the terminal row is back-tracked.

**Figure 6:**
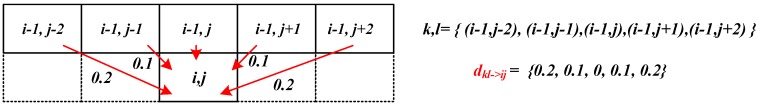
Transition to the node (i,j) is allowed only from its nearest five predecessors {k,l}. The cost of transition *d_kl__→ij_* from its predecessors is assigned empirically as [0.2, 0.1, 0, 0.1, 0.2].

The complement of the trail probability map obtained from the FCN is used to initialize the node cost of each node. Only transitions from five of node’s nearest predecessors, as shown in Figure 6, are considered valid. The transition cost *d_kl__→ij_* is empirically assigned as [0.2, 0.1, 0, 0.1, and 0.2] to penalize the transitions from distant neighbors thus favoring low-curvature trails.

The trail line is computed after backtracking the lowest cost path from the starting point towards the terminal row. A position on the terminal row where the trail terminates gives the endpoint of a local trail segment. 

**Figure 7:**
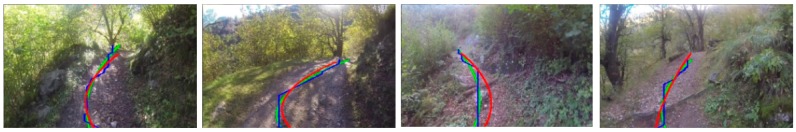
Trail detection using the proposed method. The trail detected by dynamic programming (blue color), the smoother version produced by fitting a second order polynomial (green color), and the local trail segment annotated by a human observer (red color) are superimposed on the test image.

The trail generated by DP is a coarse estimate of the trail line which at time seems unrealistic. As natural trails have low curvature, they can be coarsely approximated with e.g., low order polynomials. We assumed that 2nd order polynomial are fit to the points generated by DP to obtain a more realistic trail, as shown in Figure 7.

## 4. Experiments and Results

### 4.1. Performance of the Patch-Based Trail Classifier

**Figure 8:**
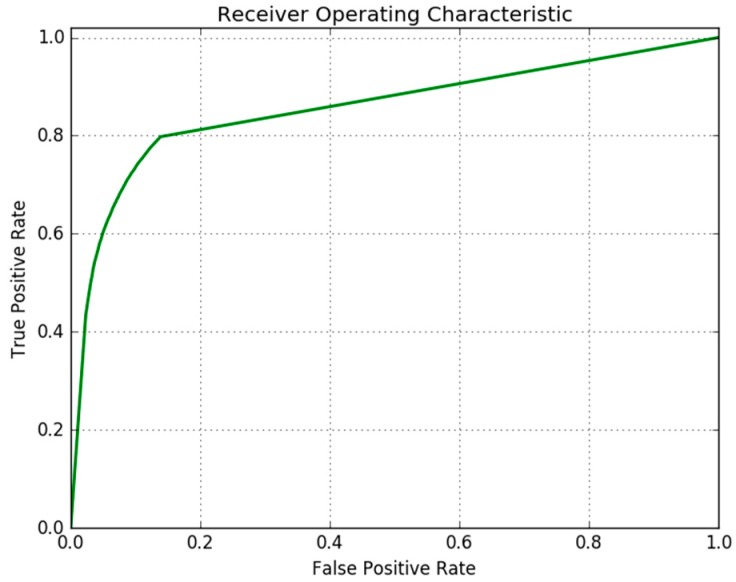
Receiver operating characteristic (ROC) curve of the DNN.

The performance of the patch-based DNN to classify trail and non-trail patches was evaluated on the testing set defined in Section 2.1. The accuracy of the patch based classifier on the testing set is 87.91%, and the confusion matrix is given in Table 1. The receiver operating characteristic (ROC) curve of the DNN is shown in Figure 8 where the area under the curve (AUC) is measured as 0.857.

### 4.2. Performance of the Trail Detection System

**Figure 9:**
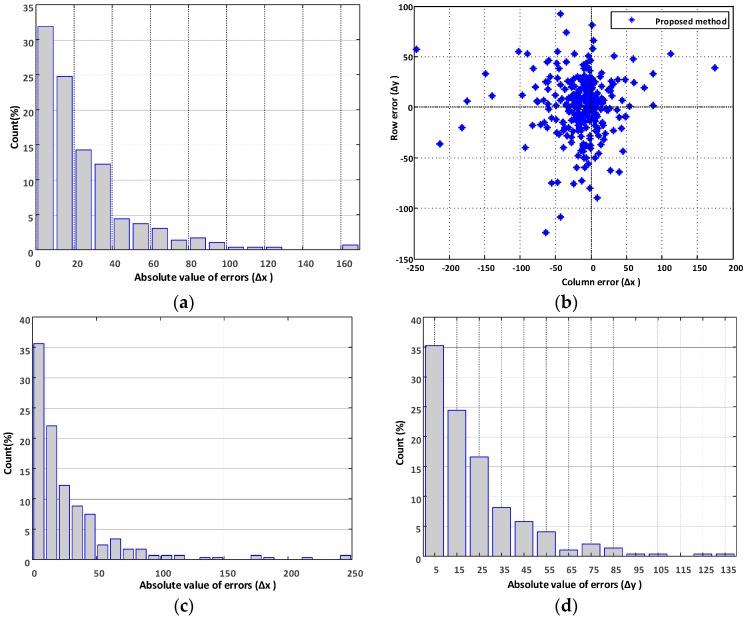
Performance of the proposed method. (**a**) Histogram of errors in determining the starting point; (**b**) The distribution of errors (Δx, Δy) in determining the endpoint; (**c**) Histogram of errors corresponding to the x component (Δx) and; (**d**) the y component (Δy) in determining the endpoint of the local trail segment.

In order to compute the accuracy of the proposed system, 294 trail images were sampled from the test subfolder GP010050 at a regular interval of five images. A human annotator was asked to mark the local trail segment visible in each of the 294 images. Examples of human marked trail segment is shown in red color in Figure 7. The error between the human annotated (starting, end) point and the corresponding points computed using the proposed system was calculated. The histogram of errors in determining the starting point horizontal coordinate (we assume starting point is at the bottom row) is as shown in Figure 9a. The distribution of errors (Δx, Δy) produced by the proposed method in determining the endpoint of the local trail is as shown in Figure 9b, and the error histograms corresponding to the x and y component of the endpoint are shown in Figure 9c,d, respectively. 

The overall accuracy of the proposed system was measured by computing the average deviation between the detected curve and the ground truth trail curve. The average pixel deviation between these two curves is computed using:

(3) $$deviation=mean\left\{\sum_{n=1}^{N}\sum_{i=1}^{L^{n}}\left|x_{i}^{d}-x_{i}^{g}\right|\right\}\;$$

where *N* is the number of images under test, *L^n^* is the length of ground truth trail curve and *x^g^* and *x^d^* are the column coordinates of the ground truth and the detected trail curves; respectively. The length of the detected trail curve is made equal to that of the ground truth curve either by extrapolation (when its length is shorter than the ground truth length) or by clamping (when its length is longer than the ground truth length).

We also measured the internal variance of human performance in the task of trail detection. We prepared another set of ground truth trail curves (for same 294 trail images) using a separate human annotator and computed the average deviation between the curves annotated by the two different annotators. The average deviation between the ground truth trail curves and the curve detected using the proposed method is presented in Table 2. 

In addition we also compare the performance of the proposed method to a modified shape template based method of [7]. We implemented a modified version of the trail detection method of [7], where we consider the output of our FCN as the most likely segmentation of the trail, instead of generating the same by grouping super-pixels. A best fitting triangle was then found by computing the shape score as suggested in [7]. The performance of this method is summarized in Table 2. 

The average deviation of 9.45 pixels between the trail curves annotated by two human annotators shows the subjective nature of the task and the challenges involved due to ambiguity between trail and non-trail areas in natural environment. The mean deviation of the proposed method averaged over the pixel deviations with the two human annotators is 23.99 pixels, whereas the average deviation for the shape guided method of [7] is worse at 26.76 pixels. 

For an input image of size 752 × 480, the overall system runs at 1 frame per second on a Intel(R) Core(TM) i7-6700K CPU 4.00 GHz (8 cores) equipped with an NVIDIA GTX980 GPU. The computations of DNN are performed on the GPU, whereas dynamic programming is implemented only on the CPU. For our unoptimized implementation, we note that 98% of the total computation time is utilized by dynamic programming. The test efficiency can be increased by a factor of four by applying dynamic programming to a down-sampled (by 2) trail-probability map without any increase in the mean pixel deviation of the resultant trail. 

### 4.3. Detecting Trail in New Environment

**Figure 10:**
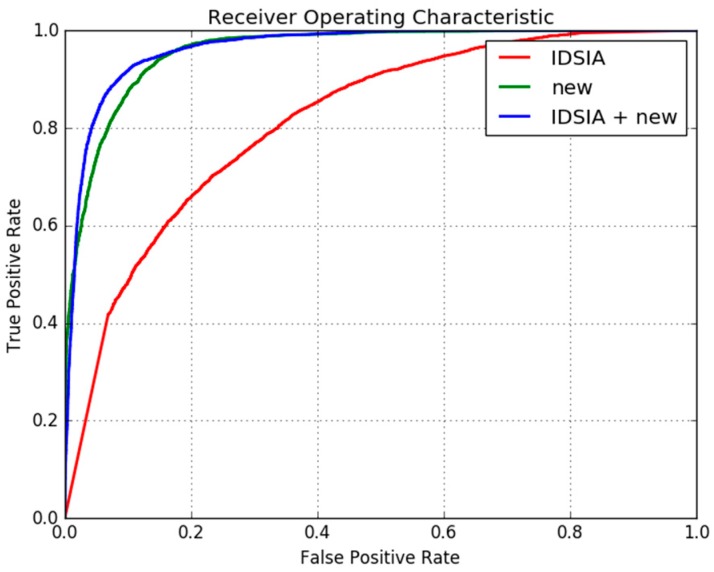
Receiver operating characteristic (ROC) curves of the network trained the IDSIA dataset, the network trained on the new data only, and the network trained on IDSIA and fine-tuned on the new data.

**Figure 11:**
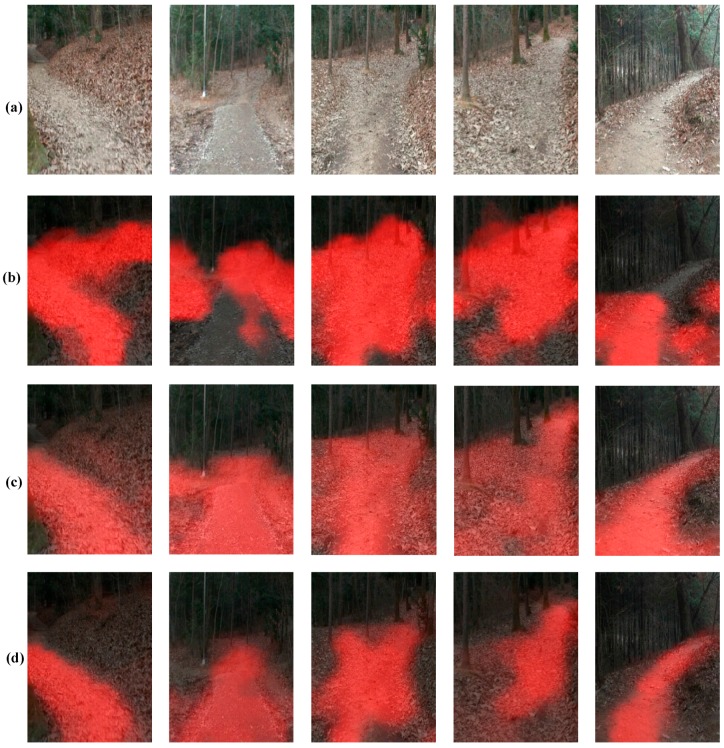
Results of trail segmentation in new environment. (**a**) Sample images from new trail; (**b**) Trail map generated by the (**b**) DNN trained on IDSIA dataset; (**c**) DNN trained on the small dataset from the new environment; (**d**) DNN trained on IDSIA dataset and fine-tuned with data from the new environment.

A disadvantage of learning-based systems is that the learning process requires a huge amount of training data. When a limited amount of training data is available, the learned system may not generalize well to scenarios not covered in the training dataset. For the application considered in this work, it is practically not possible to collect and label a huge dataset covering all the variations present in natural trails and its surrounding environment. However, a DNN trained on other similar tasks can be adapted to generalize to a new environment by fine tuning with a small amount of training data from the new environment. In this section we adapt the DNN already trained on the IDSIA dataset to detect trails on our new set of trail data. The new data was collected from a hiking trail near the city of Jeonju, South-Korea with a hand-held camcorder. The trail had varying elevation profiles and passed through light temperate deciduous forest. The images, shown in Figure 11, were captured during winter and the trail was covered with dried leaves making it completely different from the data used in Section 2.1. Only a fraction of the available data was used to adapt the DNN to this new environment. 10 random frames from a section of the trail were sampled and a total of 5038 patches of 100 × 100 pixels each were extracted from these images. 1536 patches belonged to the “trail” whereas 3502 patches belonged to the surrounding “non-trail” areas. 90% of the data was used for fine tuning the DNN and the remaining 10% was set aside for validation. Similarly, a total of 14,900 (5748 “trail” and 9152 “non-trail”) patches were extracted for testing the DNN. The parameters of this DNN already trained on the IDSIA dataset were adapted to the new trail by fine-tuning the parameters of the last two fully-connected layers only while keeping other layer parameters fixed. The confusion matrix of the DNN trained on IDSIA data and the DNN fine-tuned with the new trail data are given in Table 3 and Table 4, respectively. The accuracy of the DNN on the new testing set before fine tuning is 74.50%, whereas after fine-tuning the accuracy is increased to 90.67%. The ROC curves of network trained on the IDSIA dataset, the network trained on the new data only, and the network trained on IDSIA and fine-tuned on the new data are as shown in Figure 10. 

From Figure 10, it may seem that training only on the smaller new dataset is sufficient and fine tuning is not required, however this observation is misleading due to the limited amount of samples in the test set. A closer look at the qualitative results of trail segmentation, as shown in Figure 11, produced by the corresponding FCN’s on whole images reveal that the network trained only on the small dataset doesn’t generalize well and the fine-tuned network produces better segmentation maps.

## 5. Conclusions

The presented research has shown that deep neural networks combined with dynamic programming can be successfully applied for trail detection in natural environments. The adopted strategy of training a conventional deep neural network on small, fixed-size image chunks, followed by reshaping the network to fully convolutional architecture, capable of detailed analysis of arbitrary-sized images, proved to produce sub-optimal trail segmentation maps. Also, it has been shown that the network can be fine-tuned for recognizing novel, distinct subcategories of trails based on relatively small new training datasets. Introduction of dynamic programming on the sub-optimal segmentation maps resulted in achieving higher level trail approximations than using fixed shape templates for the trail.

The proposed method worked on single image inputs without incorporating any temporal information. However, in real world trail detection applications executed on ground-based or aerial robots, addition of temporal information could increase trail detection and trail tracking performance in several aspects. For example, confronting analysis results among consecutive frames can lead to reducing segmentation errors, and available, previous trail approximation results could speed up the forthcoming procedures.

## Figures and Tables

**Table 1:** Confusion matrix of the Deep Neural Network.

Predicted (→) Actual (↓)	Trail	Non-Trail
Trail	**11,357**	6082
Non-Trail	4562	**66,059**

**Table 2:** Mean pixel deviation between two trail curves.

Comparison	Mean Pixel Deviation
Human1-Human2	9.45
Human1-proposed method	22.7
Human2-proposed method	25.28
Human1-shape_guided [7]	25.68
Human2-shape_guided [7]	27.85

**Table 3:** Confusion matrix of the Deep Neural Network trained on the IDSIA data.

Predicted (→) Actual(↓)	Trail	Non-Trail
Trail	**3776**	1972
Non-Trail	1827	**7325**

**Table 4:** Confusion matrix of the Deep Neural Network fine–tuned with our data.

Predicted (→) Actual (↓)	Trail	Non-Trail
Trail	**4872**	876
Non-Trail	514	**8638**
